# Medical Defence Unions

**Published:** 1899-11

**Authors:** 


### MEDICAL DEFENCE UNIONS.

IN England, a Dr. Kidd, a respectable medical man, was recently sued for a sum of money by the friends of a former patient on charges of malpractice. The doctor won the suit and then entered countercharges of libel against the former plaintiff, recovering damages. The very successful termination of the affair was brought about through the agency of the Medical Defence Union of which Dr. Kidd had been a member for a number of years, keeping up his dues with regularity with no expectation of ever having to avail himself of the benefits of membership. The union conducted the defence with vigor and fidelity to the client's interest, and entirely relieved the doctor of all worry and expense. Why haven't we such an institution in this State? A lawsuit is never pleasant, but no one who has not experienced it, can appreciate how disagreeable and unsettling it is for one not only to have his skill and professional standing assailed, but to undergo the risk of being mulcted out of money.

Here comes in the great merit of a defence union, and here is where the absence of such an institution is acutely and painfully felt, for whatever be the verdict there is always the very considerable expense to the defendant of employing counsel.

Nowhere is the protection offered by an institution for defence more needed by physicians than in Atlanta. This city is the habitat of flocks of legal vultures attracted hither by the odor of much litigation over railroad accidents and the like. It is a very easy elision from a corporation to an individual, and the exposed and unprotected position of the doctor renders him a frequent object of attack and an easy mark.

Organized protection is what we need and what we could easily have if we but set about to get it. The State Medical Association or the local medical society could readily appropriate a sufficient sum of money to constitute a fund for the purpose of defending its members against unjust suits, and it is much to be hoped that a resolution to this effect shall be introduced at the next meeting of the State Association. W.

---

ATTENTION is called to the paper in this issue by Dr. Dalrymple, the State Veterinarian of Louisiana. The benefits to the public to be derived from an efficient municipal control and inspection of meat and milk, and a general supervision of the handling of food products are easily apparent. Georgia is a progressive State, and its people, if once they are enlightened on sanitary matters, such as the present, will demand and obtain the needed reforms. More expositions of the subject, such as Dr. Dalrymple has given, are all that is needed to effect the purposes advocated by him. W.

---

AN exchange which hails from the country's chiefest city, has an editorial entitled "Saranelli as a Bacteriologist." The young savant is referred to not less than half dozen times, once in connection with a long quotation and in every instance as *Saranelli*. This reminds one of the story told of Dr. McCosh, sometime president of Princeton College. Detecting a freshman in the act of creating disorder in the class-room, he cried out, shaking a denunciatory finger at the boding trembler, "I know ye, I know ye, what's your name?"

The editor knows Sanarelli but he doesn't know his name.

